# Older adults’ emotion recognition: No auditory-visual benefit for less clear expressions

**DOI:** 10.1371/journal.pone.0279822

**Published:** 2022-12-30

**Authors:** Simone Simonetti, Chris Davis, Jeesun Kim

**Affiliations:** 1 The MARCS Institute for Brain, Behaviour and Development, Western Sydney University, Sydney, Australia; 2 Brain and Mind Centre, School of Psychology, University of Sydney, Sydney, Australia; Texas A&M University, UNITED STATES

## Abstract

The ability to recognise emotion from faces or voices appears to decline with advancing age. However, some studies have shown that emotion recognition of auditory-visual (AV) expressions is largely unaffected by age, i.e., older adults get a larger benefit from AV presentation than younger adults resulting in similar AV recognition levels. An issue with these studies is that they used well-recognised emotional expressions that are unlikely to generalise to real-life settings. To examine if an AV emotion recognition benefit generalizes across well and less well recognised stimuli, we conducted an emotion recognition study using expressions that had clear or unclear emotion information for both modalities, or clear visual, but unclear auditory information. Older (n = 30) and younger (n = 30) participants were tested on stimuli of anger, happiness, sadness, surprise, and disgust (expressed in spoken sentences) in auditory-only (AO), visual-only (VO), or AV format. Participants were required to respond by choosing one of 5 emotion options. Younger adults were more accurate in recognising emotions than older adults except for clear VO expressions. Younger adults showed an AV benefit even when unimodal recognition was poor. No such AV benefit was found for older adults; indeed, AV was worse than VO recognition when AO recognition was poor. Analyses of confusion responses indicated that older adults generated more confusion responses that were common between AO and VO conditions, than younger adults. We propose that older adults’ poorer AV performance may be due to a combination of weak auditory emotion recognition and response uncertainty that resulted in a higher cognitive load.

## Introduction

In old age (e.g., over 70 years old), the ability to recognise facial or vocal expressions of emotion typically gets worse [1, 2]. Emotion recognition is a core component of social cognition that underpins favourable social interaction. As such, determining the extent of this decline in emotion recognition, and the reasons for it, is important for understanding how older adults’ social functioning may be impacted [3, 4]. Poorer emotion recognition in older age has been explained in different ways. Some researchers have adopted a neurophysiological standpoint in which poorer emotion recognition is a consequence of age-related changes in particular brain regions [5, 6]. Other proposals mainly consider cognitive, social, or motivational factors. For instance, it has been proposed that older adults are better at using effective strategies for emotion regulation [7].

The development of these theories of older adult emotion recognition is driven by discoveries about the factors that modulate older adults’ recognition performance. A good example is the finding that older adults are not uniformly poor at recognising emotion; they tend to be better at recognising a positive expression like happy and worse at recognising a negative one like angry (the positivity effect, see [8] for a recent meta-analysis; for a discussion of the flip side of this effect, the negativity effect, see [9]). This finding plays a central role in prominent motivation-based theories that explain the positivity effect in terms of older adults prioritising goals to boost the prominence of emotionally gratifying information [10]. For theories that posit age-related degradation of brain function, the positivity effect has been explained in terms of older adults’ weaker amygdala activation for emotional negative stimuli [5].

Another factor that has a clear influence on older adults’ emotion recognition, which is the focus of the current study, concerns the way that the expressions are presented. Traditionally, most research on aging and emotion recognition has used unimodal (i.e., facial or vocal) expressions as stimuli. It is only relatively recently that studies have examined multimodal emotion recognition, e.g., in which emotional expressions are both seen and heard, in an effort to have more naturalistic stimuli [11–13]. Generally, results have shown that older adults do much better with multimodal presentation of emotions to the extent that their performance level can be similar to or even better than that shown by younger adults [12]. Finding that the level of older adult’s multimodal emotion recognition of performance is like that of younger adults would most likely require a change in the theories to accommodate this. Moreover, it could be inferred from such results that older adults may not experience emotion recognition problems in real life where multimodal presentation of emotional expressions is likely to be common.

There are, however, several aspects of past studies that have used multimodal stimuli with older adults that motivate further investigation. The first concerns a general issue about the aim of previous studies. Most of these studies focussed simply on determining whether older adults receive a benefit from the combination of auditory and visual emotion information. As such they were less concerned with how such a multimodal advantage occurs.

The second related issue is that past studies have not examined older adults’ use of AV emotion information for stimuli that do not portray high-agreement stereotypical depictions of emotional expressions. That is, because previous studies of older adults’ multimodal emotion recognition largely focussed on demonstrating a multimodal benefit, they used stimuli that were conducive to showing such an effect. However, the stimuli used were not representative of the range of emotional expressions likely to be encountered in real life. For example, some studies have used a still picture combined with an auditory stimulus, rather than dynamic faces producing ongoing speech [11, 14]. Also, some studies evaluated a very small number of emotions, e.g., only two [15]. Moreover, in many studies, the auditory and visual emotion stimuli were selected to be highly recognisable [11]. Not only might this be a problem for generalising the results to daily life, where a far greater range of stimuli would be expected (see [16] for an overview of the range of AV expressive signals), but the use of unambiguous stimuli (selected to have high label agreement levels) could reduce any difference between younger and older adults by producing ceiling effects. Indeed, studies that have used more naturalistic stimuli, have produced unclear results with respect to whether multimodal presentation boosts older adult emotion to younger adult levels. For example, Cortes and colleagues [13] found that unbiased hit-rates for younger and older adults were not significantly different for auditory and visual (AV) emotion presentations (t value = 1.95, p = .053), but a lack of statistical difference does not mean the performance was equivalent. Indeed, calculating the Bayes Factor (BF_01_) for this contrast resulted in a value of 0.96, indicating that there was insufficient evidence to decide for or against a difference. Moreover, Cortes et al did not analyse how different emotions were recognised, noting the test used had only a small number of items per emotion and so was not suitable for a finer-grained analysis.

Given the above, the current study had two aims. First is to examine how older adults combine non-stereotypical auditory and visual emotion information; second, to investigate the process of AV integration in more depth than previous studies. To tackle the first aim, we tested older adults with AV expressive speech that varied in clarity of emotion information. It is well established that younger adults can use AV emotion information to achieve better performance than auditory only (AO) or visual only (VO) information alone (e.g., [17]). Moreover, it has been demonstrated that such an AV benefit even occurs when unimodal emotion information is not well recognised [18]. If older adults can get such an AV benefit, i.e., even when their recognition performance on one of the modalities is poor, then with multimodal stimuli, they should be able to achieve a recognition level closer to that of younger adults.

To investigate the process of AV integration in greater detail, we followed up Kim and Davis [18] by analysing the patterns of correct and incorrect (Note that, depending on the context, we refer to incorrect responses as confusions, competitors or non-target responses.) response data. To explain the AV benefit that young adults receive, Kim and Davis [18] examined the confusion matrices (emotions that were selected as the response when an error was made) and how the pattern of these in the unimodal (AO and VO) conditions changed in the bimodal (AV) one. In brief, they found that the confusion matrices for the different unimodal presentations of the same emotion tended to be non-overlapping, i.e., the confusions for a visually presented emotion tended not to be those made for an auditory presentation of that emotion. A confusion in the AV condition hardly ever occurred when it did not occur in both unimodal presentation conditions. Kim and Davis [18] interpreted this result in terms of how evidence for a specific emotion is evaluated from bimodal sources. For example, consider the case when “ANGRY” is presented audio-visually, it was suggested that when both sources include the same candidate emotion (e.g., “angry” in AO and VO modalities) then evidence for that interpretation is reinforced. When a candidate emotion is supported only one modality (e.g., “happy” just in AO, or “disgust” only in VO) then evidence for that emotion would be completely discounted. This evidence accumulation model explains how information from a poorly recognised expression in one modality can nevertheless boost bimodal recognition, i.e., the interpretation that is common to both modalities (“angry” in the above example) would be boosted, but unimodal confusions (unique to that modality) would have no influence.

If, with a poorly recognised unimodal emotion stimulus, older adults produced a broad range of confusions rather than the typical ones that younger adults make, then they may not get an AV benefit. That is, whereas for younger adults the confusion patterns tend to be non-overlapping, this may break down for older adults if too many candidate analyses are generated. To make this clear, consider the angry example again. If for AO presentation, older adults not only mistook “happy” but also “disgust” for auditory “ANGRY”, then, because visual only “ANGRY” is sometimes mistaken for “disgust”, then with AV presentation, the disgust interpretation would be reinforced and not eliminated. This would lead to a larger number of viable interpretations, and this could potentially undermine any AV benefit. In this regard, it is important to analyse both correct and confusion responses to understand whether and how older adults get an AV benefit for stimuli that present unclear emotion information.

As mentioned above, a limitation of many previous studies on older adults’ AV emotion recognition is that the stimuli used were selected to have high emotion recognition rates and so these stimuli would generate few alternative candidates [11, 15, 19]. As such, these stimuli cannot test how well the perceiver can use AV emotion information to winnow out candidate analyses. One study [20] did use realistic AV emotion portrayals that had both high and low recognition rates, and they found a decline in emotion recognition still occurred with increasing age. This result shows that the presentation of AV emotion information in itself does not close the gap between younger and older adult recognition performance. Interestingly, the study found that the emotions that were less well recognised showed the highest (negative) correlation between age and recognition performance, i.e., compared to younger adults, older adults had poorer emotion recognition for emotions that were not well recognised. However, as this study [20] did not use AO or VO stimuli, the results do not enable a quantification of AV benefit.

A recent study by De Boer and colleagues [21] has explicitly examined how older adults integrate auditory and visual emotion information when signals from either or both modalities have been degraded to reduce recognition performance. The study used naturalistic AV spoken emotion stimuli that presented both facial and body emotional gestures (i.e., the head and trunk of actors) from the core set of the Geneva Multimodal Emotion Portrayals (GEMEP, see [22]). De Boer and colleagues [21] found that the older participants showed approximately the same pattern of emotion recognition across degraded conditions as younger participants, i.e., older adults were as good as younger one at integrating audio and video to improve emotion recognition.

On the face of it, the De Boer et al study [21] appears to have answered the current research question about whether older adults integrate clear and less clear emotion information as well as younger adults do. However, there are two related reasons why the two studies are tackling different issues, and it is instructive to unpack these. The first reason is that [21] was specifically about the effect of sensory degradation on emotion recognition and how young and older adults compensate for this. The tacit assumptions here are that emotion renditions are typically unambiguous; and that older adults’ emotion recognition problems are due to issues in peripheral sensory processing that result in the degradation of the emotion signals. In contrast, the issue that the current study addressed was how young and old adults recognise emotions when the emotion expressions themselves differ in their clarity. In using less clear emotion stimuli, we assume that in real life not all emotion renditions are clear and that younger and older adults may differ in how well they can combine AV information for clear and less clear expressions. The second reason why [21] does not address current concerns, is to do with the method used to produce poorer emotion recognition. The De Boer et al study [21] study did an excellent job in employing realistic methods of degrading of emotion signals; methods that simulate age-related sensory problems, i.e., eye fixation linked Gaussian blur of the image to simulate age-related macular degeneration and a hearing loss simulation for audio. However, these imposed degradations would be obvious to the participants, and would act as a signal to them to give less weight to the degraded information source.

Unlike [21], the current study is interested in the case where there is no obvious degradation upon which to base the weighing of information from the sources. As such, the emotion stimuli used in the current study were selected based on the recognition results of a previous study [18] so that there was an assortment of well recognised and less well recognised stimuli. The well recognised stimuli in [18] were mostly in the visual modality and by one presenter (henceforth the clear presenter, all over 90% correct), although Disgust, Sad and Happy, had high visual recognition rates for the other (unclear) presenter too (all above 80% correct). The auditory stimuli were less well recognised in [18]: for the clear presenter, only Disgust was poorly recognised (around 50% correct); for the unclear presenter, all of the emotional expressions except Sad were poorly recognised (40–63% correct). It is this mix of clear and unclear AV spoken emotion stimuli that provide the testing ground to determine how young and older adults integrate cross-modal emotion information.

In sum, the current study examined whether older adults would benefit from AV presentation when information in one modality was unclear and if so, how the size of the AV benefit for older adults would compare with that for younger Adults and how such a multimodal advantage might have been produced. Given the previous findings [19, 20, 23], it was predicted that older adults would achieve higher levels of emotion recognition with AV stimuli than with unimodal ones for stimuli that were well recognised in each modality (i.e., stimuli from the clear presenter). Furthermore, it was predicted that for these stimuli, older and younger adults would get a similar sized AV benefit for stimuli from the clear presenter. However, based on [20], we expected that the size of the AV benefit would be smaller in older adults for ambiguous expressions (i.e., stimuli from the unclear presenter).

## Method

The study was approved by Western Sydney University Human Ethics Committee (H10938); and has been conducted according to the principles expressed in the Declaration of Helsinki. Upon arrival, each participant was given information on what the study involved (e.g., the task & duration, their right to withdraw, etc) and then informed consent was obtained in a written form.

### Participants

Thirty younger adults (Mage = 20, SD = 2.4, 20 women) from Western Sydney University participated in this study for course credit. Thirty older adults (Mage = 72, SD = 6.1, 13 women) who were recruited from local community groups (e.g., senior computer clubs) participated for monetary reimbursement. Sample sizes were determined based on [19]. All participants had learnt English at age 7 or younger. Participants were given the Mini-Mental State Exam (MMSE) [24] as the presence of dementia has been associated with poor emotion processing [25]. Younger (M = 29) and older (M = 28) adults scored within the normal range (above 23), indicating no presence of dementia. Participants had no reported history of psychiatric disorders except three older adults: one who had former PTSD and was still on antidepressant/antianxiety medicine; one had sleep apnoea; and one had migraines/headaches.

### Materials

The stimuli were selected from [18] and consisted of audio and video recordings from two men, who were native Australian English presenters (in their early twenties) uttering 8 Semantically Unpredictable Sentences [26]. In uttering each sentence, the presenters were instructed to express anger, sadness, disgust, surprise, happiness, or neutral as if they were communicating this emotion to an observer. The total 32 (2 x 8 x 6) recordings were edited to produce AO, VO, and AV stimuli. A woman presenter’s expressions were also selected for 12 practice trials.

As described previously, we selected stimuli from two presenters based on the average correct emotion recognition scores of 55 young participants from a previous study [18] (see Table 1). For the clear presenter, the emotion expressions were clearly recognisable (although this was not the case for Disgust in the AO condition). For the unclear presenter, the emotion expressions were poorly recognised in the auditory modality but produced a mixture of clear and unclear visual expressions.

**Table 1:** Mean percent correct scores (Standard Error, SE) for the clear and unclear presenters across presentation modality and emotion (data from [18]).

Clear	Anger	Disgust	Sad	Surprise	Happy
AO	97.3 (1.36)	52.8 (9.65)	85.3 (4.58)	73.4 (5.89)	81.1 (5.81)
VO	98.2 (1.48)	95.4 (3.09)	98.1 (1.55)	90.0 (4.10)	99.1 (0.91)
AV	94.5 (2.79)	97.3 (2.31)	97.3 (1.61)	94.5 (2.09)	100 (0.0)
Unclear					
AO	40.0 (7.56)	45.8 (7.73)	85.3 (7.24)	60.6 (7.45)	63.0 (5.63)
VO	46.8 (7.79)	87.0 (3.53)	82.4 (4.17)	50.5 (6.59)	81.7 (7.74)
AV	70.0 (8.37)	91.8 (2.04)	91.8 (4.67)	71.8 (7.29)	89.0 (4.78)

### Procedure

**Fig 1:**
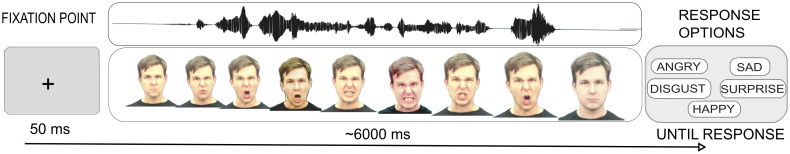
A schematic depiction of trial. Shown is an illustration of AV presentation of Angry (clear presenter) in which the middle panel represents the dynamic auditory and visual emotion information.

Participants were tested individually in a quiet room. They were told they would ‘see’, ‘hear’, or ‘see and hear’ a person conveying various emotions (while speaking) across trials. They were instructed to select one of 5 options on the screen that they thought best described the expressed emotion. As shown in Fig 1, for each trial, a fixation point was presented (50ms), followed by an experimental item (approx. 6 secs). Then, 5 boxes labelled “Angry”, “Sad”, “Disgust”, “Surprise”, and “Happy” were presented for a response (using the mouse to position the cursor over the selected option and pressing the mouse button). Participants were presented with 240 trials: three blocks (AO, VO, AV) of 40 trials (8 sentences, 5 emotions) for each of 2 presenters. Prior to each presenter block (i.e., AO, VO, AV), two neutral expressions of that presenter were displayed. Participants were told to use these expressions as a baseline to compare with subsequently presented emotions. Trials were blocked by presenter to reduce the difficulty of the task. That is, by blocking the presenter trials, as per [18], participants were given the opportunity to focus on a single presenters’ expressions and to compare emotion expressions within that presenter block (to ultimately assist emotion recognition). Practice items were presented before experimental ones.

The order of the three blocks was counterbalanced across participants. Half of the participants received the clear presenter trials first for all three blocks. The presentation order of stimuli within each presenter block was randomised. For stimulus display and response collection, we used DMDX [27].

After the experiment, hearing and visual acuity were assessed since poor acuity may unduly affect the processing of acoustic and visual signals important for emotion identification. Hearing acuity was assessed by the first author (under the supervision of an experienced audiologist) for both ears using pure tone audiometry (Diagnostic Audiometer, AD229e) for 0.5, 1, 2, and 4 kHz. The Freiburg Visual Acuity Test (FrACT) [28] was used for both eyes and, if required, participants wore corrective glasses during the eye assessment. The MMSE was also administered to participants and participants completed a questionnaire detailing age, gender, and languages spoken.

### Statistical and additional analyses

Percent correct scores were calculated (using the Emmeans R package [29]) and were analysed with a repeated measures ANOVA with modality (AO, VO, AV), presenter (clear, unclear), and emotion (angry, sad, disgust, surprise, happy) as within-participant factors, and age as a between-participants factor (see S1 Appendix for the participant data). To examine the impact of expression clarity and age on the recognition of unimodal and bimodal expressions, separate repeated measures ANOVAs were conducted for the presenters with modality and emotion as within subject factors, and age as the between-subjects factor.

#### Hearing level

Older and younger adults’ hearing level scores (as estimated by pure tone audiometry) were averaged across the right and left ears. To explore whether older adults’ hearing ability was associated with emotion recognition performance, a multiple regression analysis was performed between the recognition scores for the AO condition and hearing level for the four hearing frequencies. As per [30], we also separated the older adult participant group into two subgroups based on their hearing ability and compared them on the AO emotion recognition task.

#### Efficient AV integration

In addition to examining the possible influence of sensory factors in how well auditory and visual information are used in the selection of the correct response option, we also calculated a measure of AV integration efficiency based on the visual and auditory information by considering the pattern of errors for the visual and auditory only presentations. To gauge the efficiency to which information from the separate modalities was combined, we compared performance in the AV condition with what might be expected from the integration of the auditory and visual emotion information given by a multiplicative integration rule (essentially a Bayesian likelihood ratio). To do this, we used the full response patterns and followed [31] in which the predicted probability of a response was calculated based on the unimodal recognition performance. In this calculation, the combined AV support for the alternatives is considered; and ambiguous information (as indicated by different levels of support for auditory and visual recognition) is given less weight in the AV combination. So, for example, the probability of responding Happy given a happy auditory (*A*) presentation and a happy visual (*V*) one is given by Eq 1:

$$\frac{\left(Support\;HappyA\;\left|HappyA\;X\;Support\;HappyV\right|HappyV\right)}{\left(Support\;HappyA\;\left|HappyA\;X\;Support\;HappyV\right|HappyV\right)+\left(Support\;AngryA\left|HappyA\;X\;Support\;AngryV\right|HappyV\right)+\cdots \;remaining\;alternatives}$$ (1)

The value obtained from this formula represents the optimal combination of the AV information given the confusions that have been made to the unimodal stimuli; it will be compared with actual AV performance to determine whether the participants are efficiently combining the A and AV sources of information.

Number of candidate emotions selected: To further examine whether there was a difference between young and older adults in the efficiency of combining information from the visual and auditory modalities, we determined how many emotions other than the presented one (target) were selected for the VO, AO and AV presentations conditions (i.e., how many competitor candidates were selected).

## Results

### Age-related difference in emotion recognition

**Fig 2:**
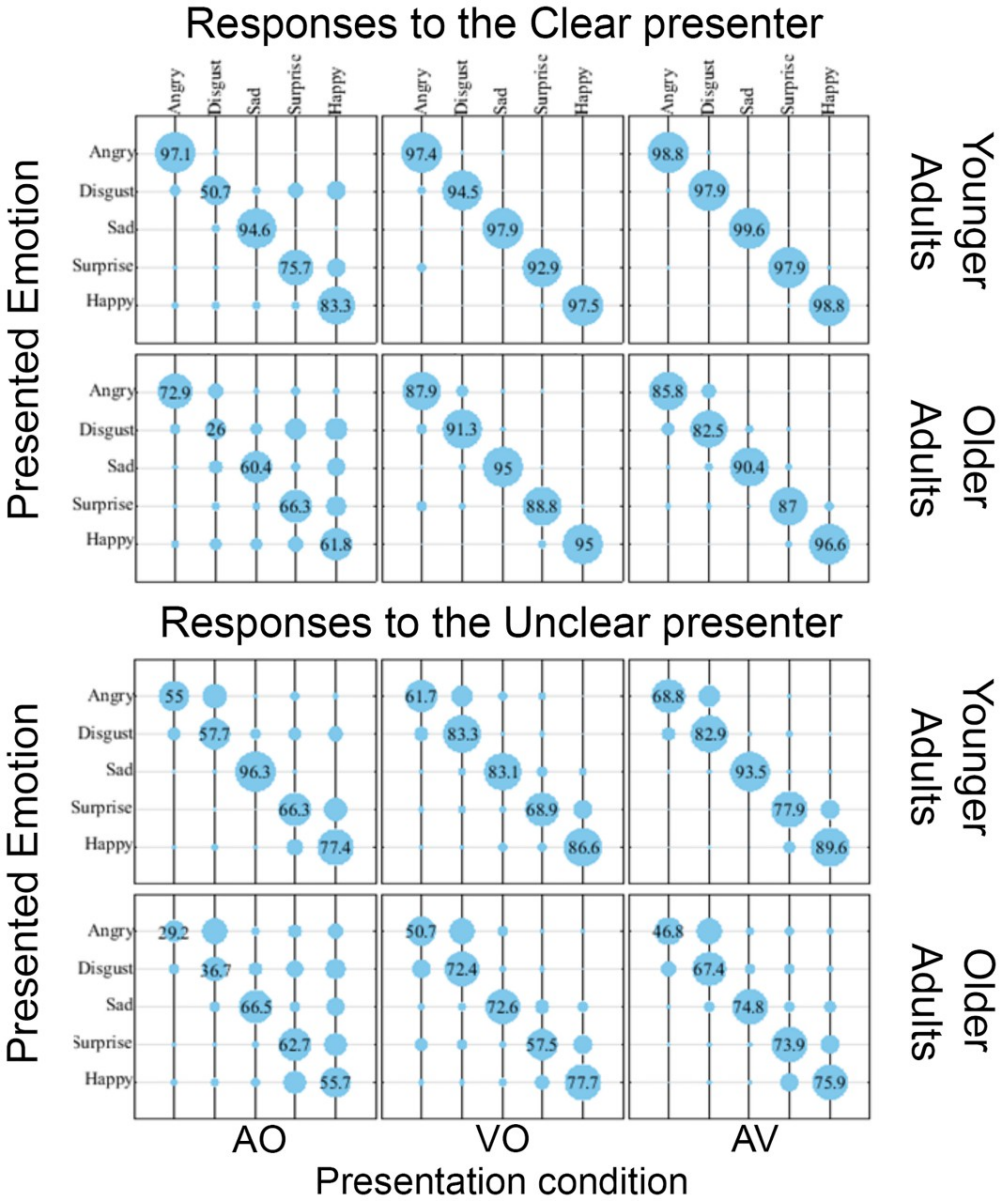
Response matrices for the AO (left column), VO (middle column), and AV (right column) presentation conditions for the Clear presenter (top two rows) and Unclear presenter (bottom two rows). Each pair of rows show the response matrices for the younger adults (top) and older adults (bottom). The presented emotions are shown on the vertical and the responses to these are shown at the top, horizontally. Response magnitude is represented by the size of the circles; the numbers reported on the diagonal have been added to indicate scale and are the mean percentage of times that a response was selected for a presented emotion.

Fig 2 provides a visual overview of the results in the form of confusion matrices that summarise the pattern of younger and older adult emotion recognition responses for the clear and unclear presenter for the Auditory only (AO), Visual Only (VO) and Auditory-Visual (AV) presentation conditions. The size of the blue circles represents the percentage of each response. For example, the large blue circled on the diagonals of each matrix represent correct recognition (with the percent response scores shown). The smaller, off-diagonal circles represent response confusions.

An analysis of response confusions is provided in the “Number of candidate emotions selected” section below. For now, it is worth pointing out that older adults have a broader spread of confusions (particularly for the AO presentations of the unclear presenter) and that for older adults, there are a greater number of confusions that are common to the AO and VO conditions. An analysis of response confusions is provided in the “Number of candidate emotions selected” section below. For now, it is worth pointing out that older adults have a broader spread of (particularly for the AO presentations of the unclear presenter) and that for older adults, there are a greater number of confusions that are common to the AO and VO conditions.

### Emotion recognition accuracy

**Fig 3:**
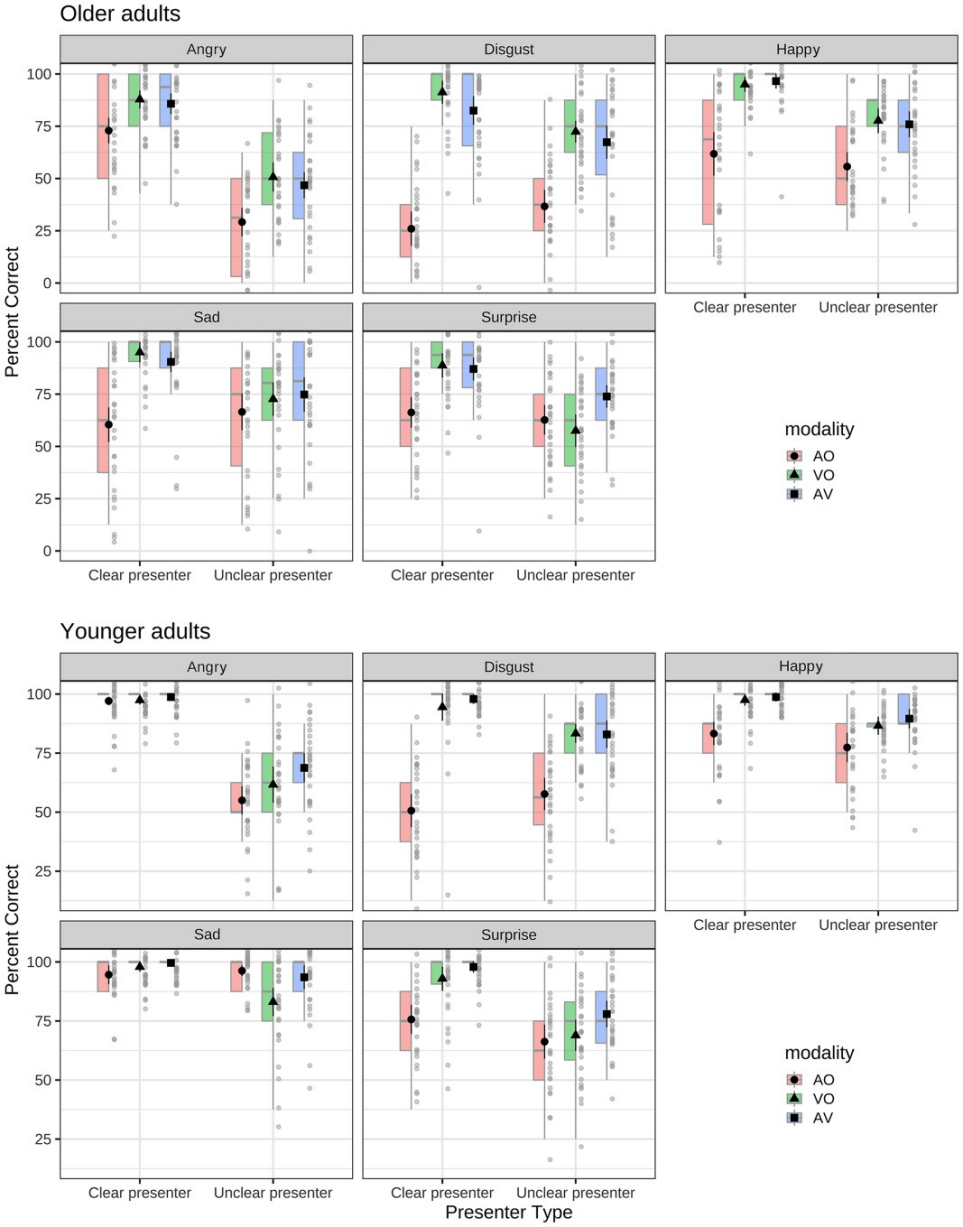
Box plots summarising the percent correct scores for older adults (top panel) and younger adults (lower panel) as a function of the clear and unclear presenter, presentation modality and emotion type. Filled symbols are the mean, horizontal bars the medium, box show the interquartile range.

Overall correct emotion recognition scores for the younger and older adults are presented in Fig 3. For younger adults, AV scores were almost always higher than the highest unimodal score as per [18]. Conversely, older adults did not show improved AV scores relative to unimodal ones; indeed, for most cases, the AV scores for older adults were numerically lower than the VO ones. This result suggests that compared to younger adults, older adults were less adept at combining auditory and visual information.

There was a significant main effect of age with younger adults (M = 84.1%, SD = 4.3, confidence limits: 81.1–87.1%) outperforming older ones (M = 70.3%, SD = 10.8, confidence limits: 67.3–73.3), *F*(1,58) = 42.76, *p* < .001, ηp2 = .42. There was also a main effect of presentation condition, with the AV (M = 84.3%, SD = 11.5, confidence limits: 81.3–87.3) and VO (M = 82.6%, SD = 8.1, confidence limits: 80.5–84.7) presentation modalities attracting the higher percent correct scores than the AO (M = 64.6%, SD = 15.2, confidence limits: 60.7–68.5%) condition, *F*(2,116) = 245.10, *p* < .001, ηp2 = .81. Participants identified the clear (M = 85.4%, SD = 10.3, confidence limits: 82.7–88.1%) presenters’ expressions better than the unclear (M = 69.0%, SD = 12.0, confidence limits: 65.9–72.1%) presenter, *F*(1,58) = 402.61, *p* < .001, ηp2 = .87. The main effect of emotion was also significant, *F*(4,232) = 37.56, *p* < .001, ηp2 = .39, showing emotion recognition scores varied as a function of emotion types. There was also a significant interaction between presentation modality, presenter, and age group, *F*(2,116) = 5.98, *p* < .01, ηp2 = .09.

For the Clear presenter, the ANOVA revealed that younger adults (M = 91.6%, SD = 4.3, confidence limits: 88.6–94.6%) outperformed older adults (M = 79.2%, SD = 10.9, confidence limits: 76.1–82.2%), *F*(1,58) = 34.06, *p* < .001, ηp2 = .37. The VO (M = 93.8%, SD = 7.5, confidence limits: 91.9–95.7%) and AV (M = 93.5%, SD = 10.7, confidence limits: 90.8–96.3%) conditions attracted the highest percent correct responses followed by the AO (M = 68.9%, SD = 16.3, confidence limits: 64.6–73.1%) condition, *F*(2,116) = 336.84, *p* < .001, ηp2 = .85. The main effect of emotion, *F*(4,232) = 37.79, *p* < .001, ηp2 = .40, was also significant. There was a significant interaction between modality, emotion, and age group, *F*(8,464) = 3.00, *p* < .01, ηp2 = .05, and this was analysed further using a Bonferroni adjusted alpha.

Post hoc analyses revealed that for expressions of disgust, older adults showed worse performance for AV relative to VO expressions (*p* < .05). For the remaining expression types (i.e., angry, sad, surprise, happy) older adults showed no performance differences when these expressions were conveyed visually or audio-visually (*p* > .32 for all comparisons). Older adults recognised AV expressions better than AO expressions across all emotion types (*p* < .001). For younger adults, there were no differences across any emotions conveyed via VO and AV modalities (*p* > .27). This was also the case for AO expressions of anger and sadness where there were no performance differences between AO and AV renditions of these emotions (*p* > .41). Only expressions of happy, surprise, and disgust were less well recognised when conveyed via the AO modality than when conveyed via the AV modality (*p* < .01). These analyses also revealed that younger adults outperformed older adults for most of the AO expressions (*p* < .01) and AV emotional expressions (*p* < .02) except for AO expressions of surprise (*p* = .07) and AV expressions of happy (*p* = .27), where older and younger groups did not show a difference. There were no differences between older and younger percent correct scores for most of the emotions in the VO presentation modality (*p* > .16); except for angry where younger adults outperformed older ones (*p* < .01).

For the Unclear presenter, the younger group (M = 76.6%, SD = 5.8, confidence limits: 73.2–80.0%) performed better than the older group (M = 61.4%, SD = 11.8, confidence limits: 58.0–64.8%), *F*(1,58) = 40.37, *p* < .001, ηp2 = .41. Participants showed better performance in the AV (M = 75.1%, SD = 13.4, confidence limits: 71.7–78.6%) and VO (M = 71.5%, SD = 11.0, confidence limits: 68.6–74.3%) modalities than the AO modality (M = 60.3%, SD = 15.4, confidence limits: 56.4–64.3%), *F*(2,116) = 74.48, *p* < .001, ηp2 = .56. The main effect of emotion was significant, *F*(4,232) = 53.73, *p* < .001, ηp2 = .48, and there was a significant interaction between modality and age group, *F*(2,116) = 7.59, *p* < .01, ηp2 = .12. This was analysed further (with a Bonferroni adjusted alpha). The interaction between modality, emotion, and age group was not significant, *F*(8,464) = 1.77, *p* = .08, ηp2 = .03.

Post hoc analyses revealed that younger adults showed better recognition of emotions when presented with AV than VO expressions, followed by AO expressions (*p* < .02). Older adults had worse recognition in the AO compared to the AV condition (*p* < .001); but showed no significant differences when recognising emotions in the VO and AV conditions (*p* > .99). These analyses also indicated that younger adults outperformed older adults across all modality types (*p* < .001 for all comparisons).

### Visual and hearing acuity and emotion recognition

**Fig 4:**
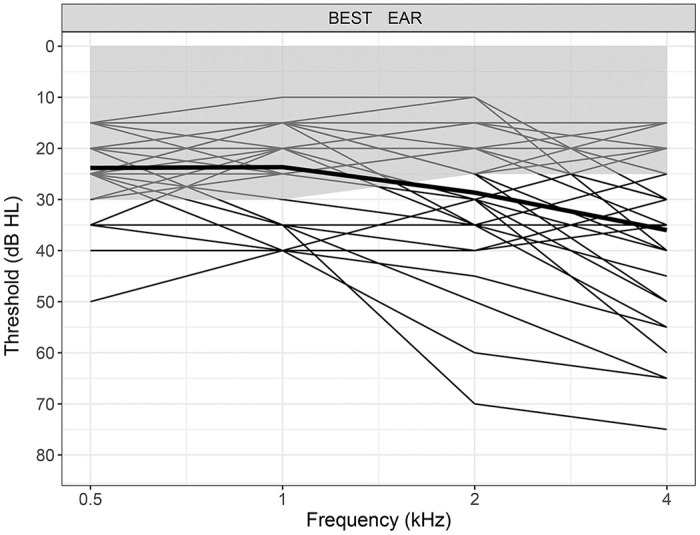
Mean hearing level (dB) dark curve and individual data for older adults across 0.5, 1, 2, and 4 kilohertz. The grey filled area shows the range of the younger adult data.

Most participants had normal or corrected-to-normal visual acuity; except one younger and three older adults who had slightly worse than normal vision (a score of 1) with scores over 0.75. As can be seen in Fig 4, older adults’ hearing level was poorer than younger adults across all frequencies. Younger adults had an averaged hearing level of 12dB across all frequencies (range = 10dB-15dB) whereas older adults averaged 32dB (range = 28dB-40dB).

A multiple regression analysis indicated that hearing levels were not significantly associated with performance for the AO emotion recognition task, adjusted *R*^2^ = .1, *F*(4,29) = 2.1, *p* = .11. When separated into two groups based on hearing ability, one group, consisting of 12 participants, had hearing levels above 30dB for 500 or 1000Hz or above 40dB for 2000Hz. The remaining 18 participants had hearing levels equal to or below these thresholds. In line with [30] these two groups did not differ in their performance for the AO emotional expressions, *F*(1,29) = 1.99, *p* = .17, η_p_^2^ = .07, providing further evidence to suggest that levels of hearing sensitivity were not associated with AO emotion recognition.

### AV integration

Table 2 shows the calculated efficient integration scores (see the “Efficient AV integration” section above) and the actual AV emotion recognition scores. There are two important aspects of the table to note, the first is the overall level of information available as given by the efficient integration score; the second is whether the actual score was different from the efficient integration score (as indicated by the asterisk). For the clear presenter, younger adults’ actual AV performance did not significantly differ from that estimated by the efficient integration calculation (and both were at ceiling). For the unclear presenter, the actual performance was on average about 6% worse and significantly different for disgust, sad and happy. The older adults’ actual AV recognition scores for the clear presenter were about 7% worse than what would be expected under efficient integration (which is at ceiling levels). For the unclear presenter, the actual recognition score was about 10% worse than the estimated optimal score, and the recognition scores for sad and happy were significantly lower than the efficient integration estimate.

**Table 2:** Mean efficient and actual AV emotion percent correct recognition scores (SD) as a function of presenter and age group.

			Angry	Disgust	Sad	Surprise	Happy
Younger Adults	Clear presenter	Efficient	100.0 (.01)	96.4 (.15)	99.9 (.00)	98.5 (.05)	99.9 (.00)
		Actual	98.8 (.40)	97.9 (.40)	99.6 (.40)	97.9 (.40)	98.8 (.40)
	Unclear presenter	Efficient	72.1 (.24)	94.7 (.10)	99.2 (.03)	81.9 (.19)	97.1 (.08)
		Actual	68.8 (.28)	82.9* (.34)	93.5* (.38)	77.9 (.31)	89.6* (.36)
Older Adults	Clear presenter	Efficient	92.9 (.12)	90.4 (.26)	98.7 (.39)	99.1 (.34)	96.0 (.12)
		Actual	85.8* (.35)	82.5 (.35)	90.4* (.37)	87.0* (.36)	96.6 (.39)
	Unclear presenter	Efficient	43.8 (.35)	75.9 (.36)	86.8 (.26)	80.3 (.22)	89.4 (.15)
		Actual	46.8 (.21)	67.4 (.31)	74.8* (.32)	73.9 (.29)	75.9* (.31)

* Actual AV scores significantly different from Predicted scores with p < .05 or less.

### Number of confusions (non-target competitor emotions selected)

The data in Table 3 shows that except the older adults’ AO condition more competitors were selected in unclear than clear conditions (*p* < .001 for all). In general, older adults selected more competitors than did younger adults (*p* < .001 for all) and this was especially the case for AO presentation. It further shows that for older adults, presenting auditory emotion expressions in addition to visual ones (i.e., AV presentation) did not reduce the number of competitors compared to only having the visual (VO) presentation (*p* > .1 for both Clear and Unclear presenters) whereas this was the case for the younger adults (*p* = .012 for the Clear presenter; p < .001 for the Unclear presenter).

**Table 3:** Mean number (Standard deviation) of other non-target (competitor) emotions selected as a function of presentation modality, presenter, and age group.

		AO	VO	AV
Younger Adults	Clear Presenter	0.99 (1.0)	0.21 (0.5)	0.1 (0.3)
	Unclear presenter	1.2 (0.88)	1.1 (0.76)	0.8 (0.67)
Older Adults	Clear Presenter	1.75 (1.06)	0.47 (0.65)	0.5 (0.784)
	Unclear Presenter	1.9 (1.02)	1.4 (0.75)	1.3 (1.0)

The number of competitors common in AO and VO (i.e., the degree of overlapping confusions across modality) was greater for older than younger adults in both clear and unclear conditions (*p* < .001 for all). In the AV conditions, the number of these competitors decreased but the difference between the age groups was maintained (*p* < .001 for all contrasts).

## Discussion

The current study investigated whether older adults would show improved emotion recognition performance for AV as compared to VO or AO presentations; and if so, whether this improvement would be of a similar size to (or even greater than) that shown by younger adults. We were particularly interested in whether older adults would show improved recognition performance for AV presentations even for stimuli where the expression in one modality was poorly recognised (as has been shown for younger adults in [18]). Whether or not older adults can efficiently combine auditory and visual emotion information to better recognise an unclear expression is relevant for gauging how well they might cope with the vagaries of real-life stimuli.

We begin by considering the results for expressions of the Unclear presenter, as this condition was a focus of the study. The results showed that overall, older adults’ recognition performance was poorer than the younger adults (M = 15.2% worse). The difference between the age groups was largest for AO presentation (M = 20.3%) and smallest for VO presentation (M = 10.5%), with the difference for AV presentation in between (M = 14.7%). It is interesting that older adults’ recognition scores with AV presentation would have been closer to those of the younger adults if they had simply ignored the concurrent auditory information (as was done by the older adults in the De Boer et al study [21] when the auditory information was artificially degraded).

In terms of AV benefit, the younger adults had better emotion recognition for AV presentations relative to emotions presented for the best unimodal condition (typically VO expressions). This AV benefit for the Unclear presenter replicated the results of [18]. In contrast, older adults did not get an AV benefit. That is, for all expressions (except surprise), AV presentation did not improve recognition performance relative to VO performance. This latter result stands in contrast to those of previous studies that have found older adults were able to benefit from AV emotion presentation, and did to the same degree, or even more so, than younger adults [19, 23].

A possible reason why older adults were unable to get an AV benefit is related to their poor AO emotion recognition. To make this point clear, we consider why older adults had poor AO emotion recognition and how this may have eliminated any AV benefit (and in one case even produced worse performance than VO presentation alone). Here, it should be pointed out that older adults’ poor AO emotion recognition did not appear to be due to reduced hearing sensitivity per se, i.e., older adults’ emotion recognition in AO condition was not correlated with their hearing acuity. This finding is consistent with [32] who showed that aiding older adult’s hearing (with hearing aids) boosted their word recognition scores (almost double, unaided accuracy was 38.1% correct; aided accuracy, 65.1% correct) but had no significant effect on their emotion recognition accuracy scores (36.0% unaided, 41.8% aided) see also [33].

We propose that older adults poor AO emotion recognition was due to their lower sensitivity to emotion cues compared to younger adults. Evidence for this comes from research that has shown that older adults tend to be less sensitive to auditory cues for emotion. So, for example, Ben-David and colleagues [34] presented younger and older adults spoken sentences that conveyed emotion (anger, fear, happiness, sadness) or were neutral and had them rate how much they agree that the sentence conveyed the predefined emotion. They found that older compared to younger adults gave lower ratings when an emotion was present. Other studies have shown similar results, with older adults less sensitive to emotion cues such as intensity or arousal, than younger adults [33]. This insensitivity to emotion cues may have less of an effect on emotion recognition performance when such cues are pronounced. However, if emotion cues are weak, then older adults’ insensitivity to emotion cues is likely to result in lower emotion recognition performance. Note that the speech of the Unclear presented has weaker cues to auditory emotion than the Clear presenter. In [35] Davis and Kim trained a classification model (logistic regression with a ridge estimator) on the utterances of the Clear and Unclear presenter respectively (as parametrised using the auditory attributes of the Interspeech 2009 emotion challenge [36]), then examined how the model could classify a hold-out set of stimuli from each presenter. It was found that the model trained on the Clear presenter’s emotional speech had better classification performance than that trained on that of the Unclear presenter, indicating that the Unclear presenter produced weaker (or inconsistent) cues to the different emotions.

We suggest that older adults’ insensitivity to emotion cues (especially to the emotional speech of Unclear presenter) had the combined effect of low AO emotion recognition and more response confusions. This is because the absence of a strong cue for an emotion could permit other interpretations of the input to be more viable. Under competitive activation-inhibition emotion models, e.g., [37], it is assumed that a strongly activated emotion representation inhibits other ones, whereas a weakly activated one allows more competitors to be active. The production of more unimodal response confusions could reduce AV emotion recognition scores in the following ways. First, more response confusions limit what can be achieved by integration. That is, poor unimodal recognition will limit what can be obtained by combining information sources (the Efficient score) because an increase in the number of confusion responses will increase the denominator of Eq 1 (see above) thus lowering the overall result. This is clear with Table 2 that shows a large difference in the Efficient integration scores between the younger and older adult groups (i.e., when comparing the younger and older adult scores in the row labelled “Efficient” in Table 2). The Efficient score represents the optimal integration of auditory and visual information (i.e., what could be obtained from the available information).

The second way that more unimodal confusions may affect AV emotion recognition scores is by limiting how well the AV integration process works. Here the effectiveness of AV integration is given by the difference between the Efficient score and the actual score. The results showed that older adults were less effective integrators than the younger adults (see Table 2). A possible reason for this is that older adults’ weaker activation of emotion cues led to more emotion candidates being considered (see Table 3) and this placed greater demands on cognitive processing. Moreover, as analysis of the confusion responses indicated, older adults had more confusion responses that were common across the AO and VO conditions than younger adults, and such confusions seem harder to overcome than those that occur in only a single modality (e.g., [18]). As such, older adults need to rely more on executive function to overcome the consequences of weaker perceptual processing. This idea is consistent with the results of a recent brain imagining study [38]. This study used auditory and visual speech stimuli with younger and older adults and found that younger adults had greater activation of auditory (superior temporal gyrus) and visual (intra-calcarine cortex) sensory related cortical regions than older adults who, in turn, had greater relative activation of dorsal regions associated with executive function. Such executive based processing may be inefficient when many perceptual candidates (competitors) have been generated.

The results for the VO and AV expressions of the Clear presenter were effectively at ceiling for both older and younger adults, and so could not be used to assess AV benefit. The results for disgust, however, differed from the rest, since for this expression older adults’ recognition performance was slightly worse when auditory information was added to the visual information. This occurred despite older adults’ VO performance being high (not different from the other expressions). One reason why AV was worse than VO recognition may be due to older adults’ very poor recognition of AO disgust (just 26% correct). Such a weak ‘signal’ for auditory disgust meant that not only did it provide little support for selecting “disgust” as a response, but that the selection and maintenance of other candidates would not have been inhibited by a clearly dominant interpretation. This lack of a dominant stimulus interpretation in the AO condition likely resulted in two things: The first is that AO confusions would potentially have reinforced VO ones; the second, is that an increased number of potential interpretations (as indexed by more confusions) would have increased cognitive load. Both factors would have led to poorer performance for AV stimuli.

### Limitations and future directions

There are several limitations to this study. First, the MMSE was used to screen out older adults who had a decline in cognitive function. Although the MMSE is commonly used as a broad guide to cognitive functioning; it may not be that sensitive in detecting milder forms of cognitive impairment [39]. Second, in the current experiment older adults were presented with the emotional expressions of younger adults. The issue of whether older adults show an own age bias in recognising emotion is undecided, with some studies producing evidence for an own age bias for some emotions [40] and other studies indicating that the face emotion expression of older adults are difficult to identify regardless of age [41]. Nevertheless, given that older adults would spend more time interacting with other of a similar age, future research should consider using emotion expressions from older faces as well. Third, multimodal recognition for non-emotional stimuli could be assessed to determine if older adults have more general problems in combining auditory and visual information. In addition, future research could also evaluate the role of executive function in efficiently combining multimodal information.

## Conclusion and implications

In sum, we found that for unclear emotion expressions, older adults unlike younger ones, did not get an AV recognition benefit. Indeed, for some emotions (e.g., disgust, and to some extent anger), AV recognition was worse than VO recognition, i.e., good VO recognition was compromised by the additional presentation of unclear AO emotion information. These findings have two main implications. First, older adults had worse unimodal emotion recognition than younger adults, and the lack of an older adult AV presentation benefit meant that with AV presentation they fell further behind the accuracy of younger adults. As such, our results do not require adjustments to theories that posit that older adults have worse emotion recognition than younger adults either due to age-related changes in motivation or brain function. Second, the multimodal emotion information typically available in everyday life, is likely not to assist older adults’ emotion recognition, since such expressions are often fleeting and non-prototypical, and so may be like the unclear emotion presentations of the current study.

To the best of our knowledge, this is the first study to test the extent that clear and unclear auditory and visual emotion information is combined by older adults. The findings highlight the potential problems faced by older adults when recognising emotion during everyday life where stereotypic emotional expressions may not be the norm. This is an important issue, since emotion recognition problems experienced by older adults can impair effective communication. Understanding the aetiology of emotion recognition problems in older adults can lead to targeted interventions to improve emotion recognition ability, and, in turn, the well-being of older adults.

## Supporting information

S1 Appendix Mean older/younger participant emotion recognition scores for the clear and unclear presenters across presentation modality and emotion. (XLSX)

## References

[1] Gonçalves AR, Fernandes C, Pasion R, Ferreira-Santos F, Barbosa F, Marques-Teixeira J. Effects of age on the identification of emotions in facial expressions: A meta-analysis. PeerJ. 2018;6:e5278. doi: 10.7717/peerj.5278 30065878 PMC6064197

[2] Ruffman T, Henry JD, Livingstone V, Phillips LH. A meta-analytic review of emotion recognition and aging: Implications for neuropsychological models of aging. Neurosci Biobehav Rev. 2008;32(4):863–81. doi: 10.1016/j.neubiorev.2008.01.001 18276008

[3] Phillips LH, Scott C, Henry JD, Mowat D, Bell JS. Emotion perception in Alzheimer’s disease and mood disorder in old age. Psychol Aging. 2010;25(1):38–47. doi: 10.1037/a0017369 20230126

[4] Schlegel K, Fontaine JR, Scherer KR. The nomological network of emotion recognition ability. Eur J Psychol Assess. 2017. doi: 10.1027/1015-5759/a000396

[5] Cacioppo JT, Berntson GG, Bechara A, Tranel D, Hawkley LC. Could an aging brain contribute to subjective well-being? The value added by a social neuroscience perspective. In: Alexander T, Susan F, Prentice D, editors. Social Neuroscience: Toward Understanding the Underpinnings of the Social Mind. Oxford Academic: Oxford University Press; 2011. pp. 249–62.

[6] Love N, Ruff G, Geldmacher D. Social cognition in older adults: a review of neuropsychology, neurobiology, and functional connectivity. Medical and Clinical Reviews. 2015;1(1):6. doi: 10.21767/2471-299X.1000006

[7] Carstensen LL, Isaacowitz DM, Charles ST. Taking time seriously: A theory of socioemotional selectivity. Am Psychol. 1999;54(3):165–81. doi: 10.1037/0003-066X.54.3.165 10199217

[8] Nasrollahi N, Jowett T, Machado L. Emotional information processing in young and older adults: meta-analysis reveals faces elicit distinct biases. Eur J Ageing. 2022;19:369–79. doi: 10.1007/s10433-021-00676-w 36052179 PMC9424464

[9] Simonetti S, Davis C, Kim J. Older adults get masked emotion priming for happy but not angry faces: Evidence for a positivity effect in early perceptual processing of emotional signals. Cogn Emot. 2022. doi: 10.1080/02699931.2022.2138269 36300438

[10] Reed AE, Carstensen LL. The theory behind the age-related positivity effect. Front Psychol. 2012;3:339. doi: 10.3389/fpsyg.2012.00339 23060825 PMC3459016

[11] Chaby L, Boullay VL-d, Chetouani M, Plaza M. Compensating for age limits through emotional crossmodal integration. Front Psychol. 2015;6:691. doi: 10.3389/fpsyg.2015.00691 26074845 PMC4445247

[12] Sze JA, Goodkind MS, Gyurak A, Levenson RW. Aging and emotion recognition: not just a losing matter. Psychol Aging. 2012;27(4):940–50. doi: 10.1037/a0029367 22823183 PMC3746016

[13] Cortes DS, Tornberg C, Bänziger T, Elfenbein HA, Fischer H, Laukka P. Effects of aging on emotion recognition from dynamic multimodal expressions and vocalizations. Sci Rep. 2021;11(2647):1–12. doi: 10.1038/s41598-021-82135-1 33514829 PMC7846600

[14] De Gelder B, Vroomen J. The perception of emotions by ear and by eye. Cogn Emot. 2000;14(3):289–311. doi: 10.1080/026999300378824

[15] Wieck C, Kunzmann U. Age differences in emotion recognition: A question of modality? Psychol Aging. 2017;32(5):401–11. doi: 10.1037/pag0000178 28581309

[16] Kim J, Bailly G, Davis C. Introduction to the special issue on auditory-visual expressive speech and gesture in humans and machines. Speech Commun. 2018;98:63–7. doi: 10.1016/j.specom.2018.02.001

[17] Paulmann S, Pell MD. Is there an advantage for recognizing multi-modal emotional stimuli? Motiv Emot. 2011;35(2):192–201. doi: 10.1007/s11031-011-9206-0

[18] Kim J, Davis C. Perceiving emotion from a talker: How face and voice work together. Vis Cogn. 2012;20(8):902–21. doi: 10.1080/13506285.2012.713874

[19] Hunter EM, Phillips LH, MacPherson SE. Effects of age on cross-modal emotion perception. Psychol Aging. 2010;25(4):779–87. doi: 10.1037/a0020528 21186914

[20] Schlegel K, Grandjean D, Scherer KR. Introducing the Geneva emotion recognition test: an example of Rasch-based test development. Psychol Assess. 2014;26(2):666–72. doi: 10.1037/a0035246 24295238

[21] de Boer MJ, Jürgens T, Başkent D, Cornelissen FW. Auditory and Visual Integration for Emotion Recognition and Compensation for Degraded Signals are Preserved With Age. Trends Hear. 2021;25:1–20. doi: 10.1177/23312165211045306 34617829 PMC8642111

[22] Bänziger T, Mortillaro M, Scherer KR. Introducing the Geneva Multimodal expression corpus for experimental research on emotion perception. Emot. 2012;12(5):1161–79. doi: 10.1037/a0025827 22081890

[23] Lambrecht L, Kreifelts B, Wildgruber D. Age-related decrease in recognition of emotional facial and prosodic expressions. Emot. 2012;12(3):529–39. doi: 10.1037/a0026827 22251048

[24] Folstein MF, Folstein SE, McHugh PR. “Mini-mental state”: a practical method for grading the cognitive state of patients for the clinician. J Psychiatr Res. 1975;12(3):189–98. doi: 10.1016/0022-3956(75)90026-6 1202204

[25] Torres Mendonça De Melo Fádel B, Santos De Carvalho RL, Belfort Almeida Dos Santos TT, Dourado MCN. Facial expression recognition in Alzheimer’s disease: A systematic review. J Clin Exp Neuropsychol. 2019;41(2):192–203. doi: 10.1080/13803395.2018.1501001 30088784

[26] Benoît C, Grice M, Hazan V. The SUS test: A method for the assessment of text-to-speech synthesis intelligibility using Semantically Unpredictable Sentences. Speech Commun. 1996;18(4):381–92. doi: 10.1016/0167-6393(96)00026-X

[27] Forster KI, Forster JC. DMDX: A Windows display program with millisecond accuracy. Behav Res Methods Instrum Comput. 2003;35(1):116–24. doi: 10.3758/bf03195503 12723786

[28] Bach M. The Freiburg Visual Acuity Test-variability unchanged by post-hoc re-analysis. Graefes Arch Clin Exp Ophthalmol. 2006;245(7):965–71. doi: 10.1007/s00417-006-0474-4 17219125

[29] Russell L. emmeans: estimated Marginal Means, aka Least-Squares Means. R package version 1.4. 3.01. The University of Iowa Iowa City, IA; 2019.

[30] Orbelo DM, Grim MA, Talbott RE, Ross ED. Impaired comprehension of affective prosody in elderly subjects is not predicted by age-related hearing loss or age-related cognitive decline. J Geriatr Psychiatry Neurol. 2005;18(1):25–32. doi: 10.1177/0891988704272214 15681625

[31] Massaro DW, Cohen MM. Tests of auditory–visual integration efficiency within the framework of the fuzzy logical model of perception. J Acoust Soc Am. 2000;108(2):784–9. doi: 10.1121/1.429611 10955645

[32] Goy H, Pichora-Fuller MK, Singh G, Russo FA. Hearing aids benefit recognition of words in emotional speech but not emotion identification. Trends Hear. 2018;22. doi: 10.1177/2331216518801736 30249171 PMC6156210

[33] Dupuis K, Pichora-Fuller MK. Aging affects identification of vocal emotions in semantically neutral sentences. J Speech Lang Hear Res. 2015;58(3):1061–76. doi: 10.1044/2015_JSLHR-H-14-0256 25810032

[34] Ben-David BM, Gal-Rosenblum S, van Lieshout PH, Shakuf V. Age-related differences in the perception of emotion in spoken language: The relative roles of prosody and semantics. J Speech Lang Hear Res. 2019;62: 1188–1202. doi: 10.1044/2018_JSLHR-H-ASCC7-18-0166 31026192

[35] Davis C, Kim J. Auditory and Visual Emotion Recognition: Investigating why some portrayals are better recognized than others. In Proc. The 15th International Conference on Auditory-Visual Speech Processing. 2019; pp. 33–37.

[36] Schuller B, Steidl S, Batliner, A. The interspeech 2009 emotion challenge. In Proc. The Interspeech. 2009.

[37] Suri G, Gross JJ. (2022). What is an emotion? A connectionist perspective. Emot Rev. 2022;14(2): 99–110. doi: 10.1177/17540739221082203

[38] Diaz MT, Yalcinbas E. The neural bases of multimodal sensory integration in older adults. Int J Behav Dev. 2021;45(5):409–17. doi: 10.1177/0165025420979362 34650316 PMC8514160

[39] Aggarwal A, Kean E. Comparison of the Folstein Mini Mental State Examination (MMSE) to the Montreal Cognitive Assessment (MoCA) as a cognitive screening tool in an inpatient rehabilitation setting. Neurosci Med. 2010;1(2):39–42. doi: 10.4236/nm.2010.12006

[40] Campbell A, Murray JE, Atkinson L, Ruffman T. Face age and eye gaze influence older adults’ emotion recognition. J Gerontol B Psychol Sci Soc Sci. 2017;72(4):633–6. doi: 10.1093/geronb/gbv114 26721879

[41] Riediger M, Voelkle MC, Ebner NC, Lindenberger U. Beyond “happy, angry, or sad?”: Age-of-poser and age-of-rater effects on multi-dimensional emotion perception. Cogn Emot. 2011;25(6):968–82. doi: 10.1080/02699931.2010.540812 21432636

